# Gastric adenomas in familial adenomatous polyposis are common, but subtle, and have a benign course

**DOI:** 10.1186/1897-4287-12-4

**Published:** 2014-02-24

**Authors:** Saowanee Ngamruengphong, Lisa A Boardman, Russell I Heigh, Murli Krishna, Maegan E Roberts, Douglas L Riegert-Johnson

**Affiliations:** 1 Division of Gastroenterology and Hepatology, Mayo Clinic, 4500 San Pablo Road, Jacksonville, FL 32224, USA; 2 Division of Gastroenterology and Hepatology, Mayo Clinic, Rochester MN, USA; 3 Division of Gastroenterology and Hepatology, Mayo Clinic, Scottsdale AZ, USA; 4 Department of Pathology, Mayo Clinic, Jacksonville FL, USA; 5 Division of Medical Genetics, Mayo Clinic, Jacksonville FL, USA

**Keywords:** Gastric adenoma, Stomach neoplasm, Familial adenomatous polyposis

## Abstract

**Background:**

Patients with familial adenomatous polyposis (FAP) are known to have an increased risk for gastric adenomas. The clinical features of gastric adenomas in FAP have not been well characterized, and there is a lack of standardized approaches to the management of these lesions.

**Aims:**

To study the endoscopic appearance, risk factors, clinical course, and response to therapy of gastric adenomas in patients with FAP.

**Methods:**

We retrospectively reviewed the records of 97 patients with FAP who underwent esophagogastroduodenoscopy (EGD) at Mayo Clinic (Florida, Rochester and Arizona) between 2004 and 2013.

**Results:**

Nine patients (9%) had biopsy-proven gastric adenomas. Adenomas were located in the antrum (five patients), in the body and fundus in the setting of background fundic gland polyps (FGP) (three patients), and in the body not associated with FGP (one patient). Adenoma size was 3–40 mm and the number of adenomas per patient ranged from one to 20. Adenomas in the antrum were flat and subtle, whereas those in the gastric body or fundus were polypoid and difficult to differentiate from the cystic FGPs seen in patients with FAP. The performing endoscopists reported difficulty with identifying adenomas, and six patients had at least one EGD within the previous three years where gastric adenomas were not reported. Adenomas were classified as tubular in eight patients and tubulovillous in one patient. High grade dysplasia was noted in one patient. After a median follow-up of 63 months (interquartile range: 20–149 months), no patient in our entire cohort (with or without gastric adenomas) developed gastric cancer. The patients in whom gastric adenoma developed, compared to those without gastric adenoma, were more likely to be younger [36 ± 12 vs. 48 ± 15 years, p = 0.02], have concomitant chronic gastritis [22% vs. 0%, p = 0.008], and have desmoid tumors [5 (56%) vs. 19 (22%), p = 0.04].

**Conclusions:**

Gastric adenomas are not uncommon in patients with FAP and are often difficult to identify endoscopically. Endoscopists should have a high degree of suspicion for gastric adenomas in these patients and a low threshold to biopsy. Given the benign clinical course, recommended initial management is conservative with endoscopic therapy and periodic surveillance.

## Background

Familial adenomatous polyposis (FAP) is an inherited autosomal-dominant disease primarily characterized by the development of colorectal adenomas and carcinomas. Patients with FAP may also secondarily develop duodenal, gastric, and thyroid neoplasia, as well as desmoid tumors [1]. Recently, with the widespread application of endoscopic and genetic screening with prophylactic colon surgery [2], the incidence of colorectal cancer has declined in FAP patients. As a result, the long-term survival of FAP patients is now expected to be determined by the development of extracolonic neoplasia [3]. While duodenal and thyroid neoplasia and desmoid tumors have been well studied in FAP patients, detailed clinical data on gastric adenomas is lacking.

The prevalence of gastric adenomas in FAP was about 10% in a series from the United States and ranged from 36-50% in three studies from Asia [4-6]. It is unclear if gastric adenomas in FAP patients confer an increased risk for carcinogenesis through the adenoma-carcinoma sequence or another pathway. Risk factors for the development of gastric adenomas in the setting of FAP, their typical endoscopic appearance, and the appropriate treatment are not clearly defined. We performed a retrospective review of FAP patients who had undergone esophagogastroduodenoscopy (EGD) in our institution to optimize the diagnostic and clinical management of these lesions.

## Methods

We identified 97 FAP and attenuated FAP patients who had had an EGD between January 2004 and December 2012 at Mayo Clinic (Arizona, Florida and Minnesota). Patients were identified by retrospective review of several clinical databases. All patients had a diagnosis of FAP or attenuated FAP established by accepted criteria utilizing personal and family history, clinical examination, histopathological assessment, and *APC* gene mutation testing. The study was approved by the Mayo Clinic Institutional Review Board.

For each patient, data was collected including age, gender, family history of gastric cancer, the presence of cystic fundic gland polyps (FGP), gastric adenomas, and duodenal adenomas with severity staged using the modified Spigelman classification [7], the presence of desmoid tumors, smoking history, and use of nonsteroidal anti-inflammatory drugs (NSAID) and/or proton pump inhibitors (PPI). In patients with gastric adenomas, we retrieved data on endoscopic and histological characteristics, type of directed therapy, procedure-related complications, development of cancer, and length of follow-up.

### Statistical analyses

The Pearson chi-square test or Fisher exact test was used to assess the univariate associations between a categorical variable and the presence of gastric adenoma. The student t test was used for age. A two-tailed p-value was used in all the analyses. A p-value < 0.05 was considered statistically significant. All data were analyzed using SPSS Statistics software (version 16; IBM Corporation, USA).

## Results

Nine of 97 patients with FAP (9.2%) had biopsy-proven gastric adenomas. Mean age at the time of diagnosis with a gastric adenoma was 36 years (range 18–52 years). The median follow-up for the entire cohort was 63 months (interquartile range: 20–149 months). High-grade dysplasia (HGD) was found in one patient (Patient 7) at their initial diagnosis of gastric adenoma (Table 1).

**Table 1:** Clinical characteristics of familial adenomatous polyposis patients with gastric adenomas

**Patient**	**Age/gender**	**Type of lesion**	**Site of polyp**	**Number of adenoma**	**Largest size (mm)**	**Histology**	**Treatment × treatment sessions**	**Duration of endoscopic follow up (months)**	**Post treatment outcome**
**1**	52 F	Flat	Antrum	1	NA	Tubular	CBF × 1	9	No residual adenoma at last EGD
**2**	24 M	Sessile	Body in the background of FGP	Multiple	40	Tubulovillous	Endoscopic mucosal resection × 1	3	Residual adenoma
**3**	51 F	Sessile	Antrum	3	10	Tubular	CBF × 1	11	Pending repeat EGD
**4**	18 M	Pedunculated and sessile	Fundus in the background of FGP	Multiple	5	Tubular	CBF × 1	12	Pending repeat EGD
**5**	39 M	Flat and sessile	Antrum	20	8	Tubular	CBF × 2	19	Residual adenoma
**6**	51 F	Sessile	Body	Multiple	3	Tubular	CBF × 4	109	No residual adenoma at the last EGD
**7**	38 F	Sessile	Antrum	>13	15	Tubular with HGD	Hot snare × 3	11	HGD was present at the last EGD. Death due to desmoid tumor.
**8**	32 F	NA	Body in the background of FGP	Multiple	4	Tubular	CBF × 2	65	No residual adenoma at last EGD
**9**	24 M	Sessile	Antrum, cardia	2	6	Tubular	CBF × 1	23	No residual adenoma at last EGD

CBF: Cold biopsy forceps; EGD: esophagogastroduodenoscopy; FGP: fundic gland polyps; HGD: high-grade dysplasia; NA: not available.

**Figure 1:**
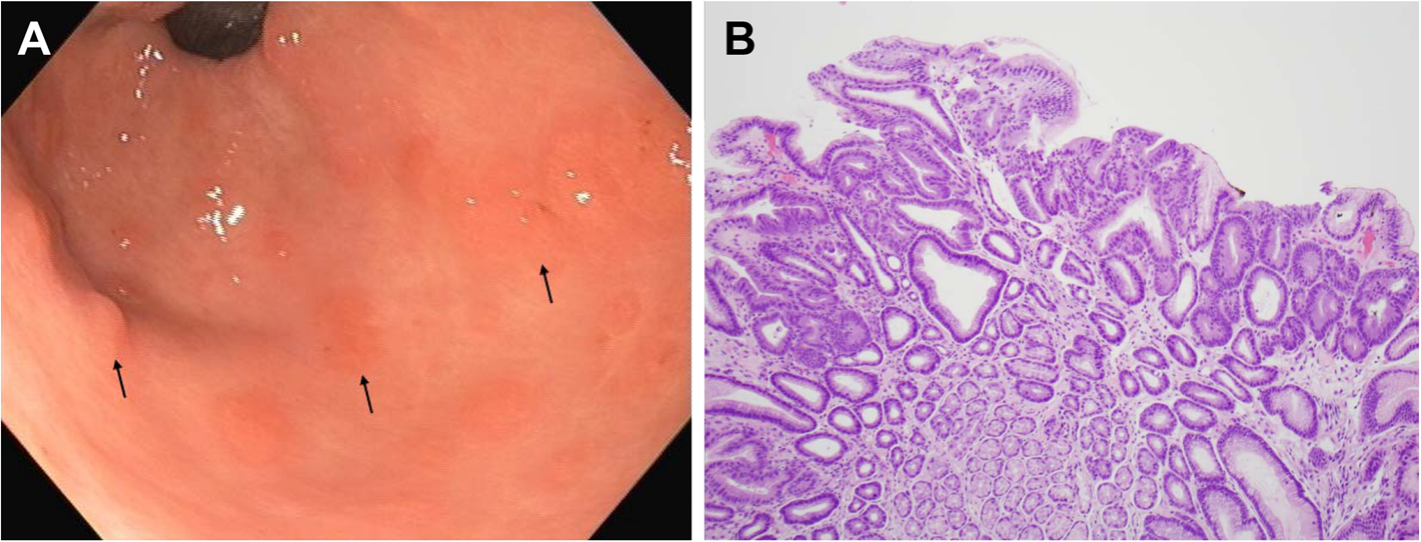
**Patient 5. (A)** Several small sessile tubular adenomas in the gastric antrum of a 39-year-old male patient with familial adenomatous polyposis (arrows). The adenomas have a flat-topped, villiform appearance. **(B)** A photomicrograph of a gastric adenoma from this patient demonstrates a tubular adenoma with low grade dysplasia, characterized by nuclear hyperchromasia and glandular crowding (H&E stain, magnification × 100).

**Figure 2:**
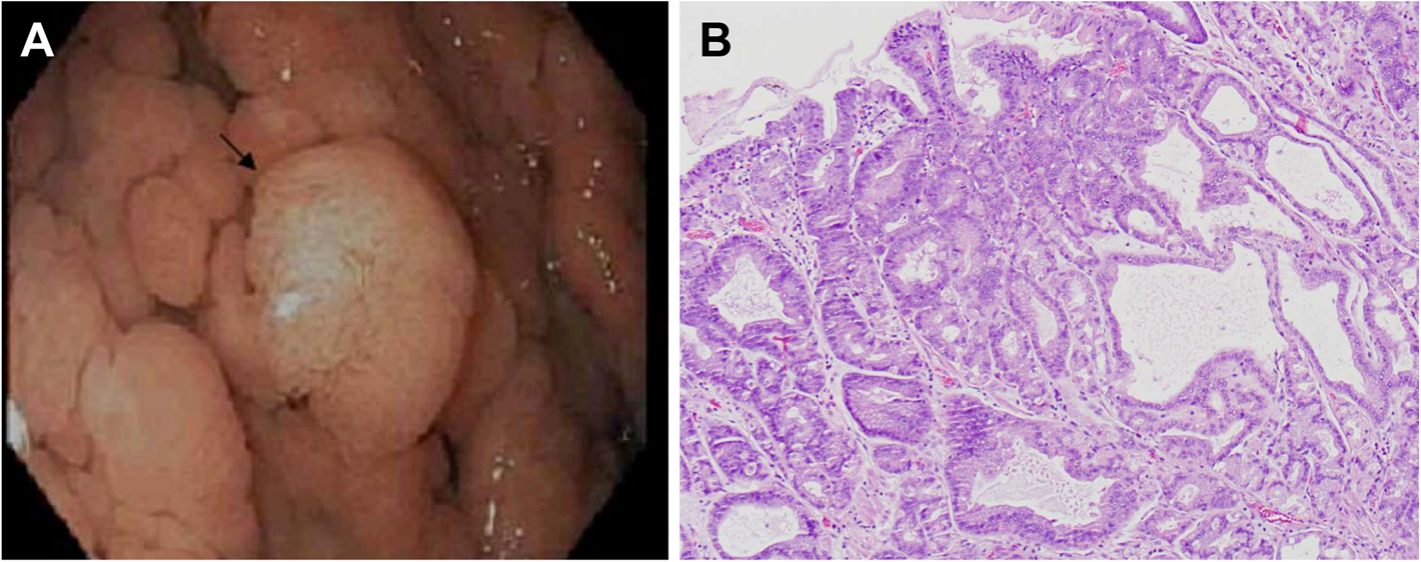
**Patient 2. (A)** A pedunculated adenoma, about 4 cm in diameter was found in a 24-year-old male patient with familial adenomatous polyposis. The lesion has a lobular, pale yellow surface and arises from a background of cystic fundic gland polyps in the gastric body. **(B)** A photomicrograph of the same polyp showing an adenoma with low grade dysplasia associated with an underlying cystic fundic gland polyp (H&E stain, magnification × 100).

Of the nine patients with gastric adenomas, four had adenomas located in the gastric antrum, three had adenomatous polyps associated with FGP, one had adenomas in the gastric body without underlying FGP, and one patient had adenomas in the gastric antrum and the gastric cardia. Gastric adenomas ranged in size from 3-40 mm and the number of adenomas ranged from one to 20 per patient. Adenomas in the antrum were flat, sessile, and subtle with a villiform red appearance, whereas those in gastric body or fundus were polypoid, lobular with pale yellow surface, and difficult to differentiate from the benign FGP seen in patients with FAP (Figures 1 and 2). A majority of performing endoscopists reported that the adenomas were difficult to identify. Six patients had at least one EGD within the previous three years with no reported endoscopic findings suggesting adenomas. Histology was tubular in eight patients and tubulovillous in one patient. None of the nine patients with gastric adenoma had Helicobacter pylori.

Various endoscopic techniques were performed for the diagnosis and treatment of gastric adenomas (Table 1). Seven of nine patients had follow-up EGD (all except Patients 3 and 4). Four of seven patients (Patients 1, 6, 8, 9) had no residual adenoma. These patients had relatively small adenomas (3–6 mm) and all were treated with cold biopsy forceps. Three patients (Patients 2, 5, 7) with numerous (13 to more than 20) and larger size of adenomas (8–40 mm) had residual adenoma at follow-up EGD. Patient 7, with multiple antral gastric adenomas containing HGD, underwent three endoscopic resections with hot snare and the most recent follow-up biopsies still showed HGD. This patient died from complications of a desmoid tumor. All other patients were alive at last follow up. After a median follow-up of 12 months (interquartile range 10–44 months) after initial gastric adenoma diagnosis, none of our patients had a progression to more advanced neoplasia from baseline pathology, including development of gastric cancer. None of the patients experienced procedure-related complications.

The FAP patients in whom gastric adenomas developed, as compared to those without gastric adenomas, were more likely to be younger [36 ± 12 vs. 48 ± 15 years, p = 0.02], have concomitant chronic gastritis [2 (22%) vs. 0 (0%), p = 0.008], and have desmoid tumors [5 (56%) vs. 19 (22%), p = 0.04]. We found no significant difference in gender, family history of gastric cancer, smoking, the presence of FGP or duodenal adenoma, the proportion of patients with modified Spigelman’s grade III or IV duodenal polyposis, and NSAID and proton pump inhibitor use between patients with and without gastric adenomas (Table 2).

**Table 2:** Characteristics of familial adenomatous polyposis patients with and without gastric adenomas (n = 97)

**Variables**	**Gastric adenoma present (n = 9)**	**No gastric adenoma (n = 88)**	**p value**
**Age: range +/- SD; yr**	36 +/- 12	48 +/- 15	0.02
**Male (%)**	4 (44%)	37 (42%)	1.00
**Race**			0.05
White	6 (67%)	81 (92%)	
Non-white	3 (33%)	7 (8%)	
**Family history of gastric cancer**	0 (0%)	3 (3%)	0.85
**Fundic gland polyps**	7 (78%)	68 (77%)	1.00
**Chronic gastritis**	2 (22%)	0 (0%)	0.01
**Any duodenal adenoma**	7 (78%)	67 (76%)	1.00
**Modified Spigelman’s grade III or IV duodenal polyposis**	4 (44%)	24 (27%)	0.27
**Desmoid tumor**	5 (56%)	19 (22%)	0.04
**NSAID or COX2 inhibitor use**	4 (44%)	32 (36%)	0.85
**Proton pump inhibitor use**	4 (44%)	25 (28%)	0.58

## Discussion

Gastric adenomas are of concern because of their suspected potential to transform into gastric cancer. The risk of gastric cancer in FAP was reported to be increased in Korean patients, with a standardized incidence ratio of 6.9 (95% confidence interval 1.4-20.1) [8]. This increased risk may be attributable to the high background risk of gastric cancer in the Korean population. Only a few cases of gastric cancer have been reported in Western FAP patients [9-11]. In this series, with a median of five years of follow-up, no patient with or without a gastric adenoma was diagnosed with gastric cancer. In 1992, another American FAP center also reported that FAP patients were not at an increased risk for gastric cancer compared to the general population [12]. A review of all of the evidence, shows no indication that Western FAP patients with or without gastric adenomas are at higher risk than the general population for gastric cancer. For definitive confirmation, a population based study of a large number of FAP patients would be needed.

In the current study, gastric adenomas were detected in about 9% of patients with FAP, similar to another study of an American population [4]. This is the first report of detailed endoscopic data, risk factor analysis, and follow-up data of gastric adenomas in a Western FAP population. Our study has shown that adenomas tend to be multiple and commonly occur in the gastric antrum, but can arise in other areas of stomach. Small gastric adenomas are likely to be successfully managed by endoscopic treatment. While endoscopic therapy for large and/or multiple lesions seems to be associated with risk of residual adenoma, none of these patients had a progression to advanced neoplasia from baseline pathology. One did have HGD at baseline, which indicates at least some potential for progression.

The tendency to develop multiple gastric adenomas in FAP patients in our study was similar to a report of Japanese FAP patients [5,13]. Gastric adenomas had one of three different presentations: flat or sessile adenomas in the gastric antrum (most common); “de novo” adenomatous polyps in the gastric body/fundus; and adenomatous changes arising from FGP. The latter two forms were difficult to separate in the patients who had numerous FGP. Three of nine patients in our study had adenomas in the gastric body/fundus that arose in a background of numerous (carpet-like) FGP. It is possible that these adenomas arose from benign FGP. In one Italian study, adenomatous or dysplastic changes within FGP have been reported in up to 44% of FAP patients [14]. Similarly, a study in American FAP patients found that dysplasia was detected in 41% of patients with FGP [4].

We noted that six of nine patients with gastric adenomas had at least one EGD within the previous three years where gastric adenomas were not reported. Since these adenomatous lesions can be subtle and difficult to differentiate from a carpet of FGP, in some cases adenomas most likely had been overlooked during previous endoscopies. In studies of non-FAP patients, the gastric adenoma/cancer miss rate by EGD was 11-19% [15,16]. The majority of missed lesions were due to endoscopist oversight [17]. In this study of FAP patients, the miss rate appears to be even higher.

The optimal management for gastric adenomas in Western FAP patients is unclear due to a paucity of data regarding prognosis and outcomes of therapy. Iida, et al. [5] described the natural history of gastric adenomas in Japanese patients with FAP. They concluded that gastric adenomas rarely changed over a long periods of time. One of 13 patients with gastric adenomas developed gastric cancer. This patient had one gastric adenoma that did not change in size and morphology during four years of follow-up, but malignant foci was confirmed by endoscopic biopsy. In our series, one patient had a tubular adenoma with HGD, but none had carcinoma. After endoscopic treatment and close follow-up EGD, progression to more advanced neoplasia from baseline pathology was not seen in any patients. Given the absence of malignancy and the response to endoscopic therapy described in our study, we propose endoscopic management with careful periodic surveillance examinations as the preferred treatment for gastric adenomas in FAP patients. Surgery should be reserved for patients with adenomas having advanced histologic features who fail endoscopic management.

*APC* mutation data was only available for three patients with gastric adenomas, which is not enough to make any comment on a possible genotype/phenotype relationship. However, there is a well described genotype/phenotype relationship in FAP for 3′ *APC* mutations and desmoids tumors [18]. By extension, a possible relationship between 3′ mutations and gastric adenoma has been suggested, as we have noted, there is a significantly higher prevalence of desmoid tumors in FAP patients with gastric adenomas than those without gastric adenomas. Spigelman et al. [19] reported a high incidence of gastric adenomas in patients with severe duodenal polyposis. In their study, five of six patients with gastric adenomas had stage III or IV duodenal disease. In our study, we found no association between the presence of gastric adenomas and advanced duodenal polyposis. Although chronic gastritis was associated with gastric adenomas, H. pylori infection was not found in any of these patients. Review of the records of patients with gastric adenomas also did not identify any indication of autoimmune gastritis such as vitamin B12 deficiency or hypothyroidism. Although H. pylori was not found in the gastric biopsies of patients with chronic gastritis, it is possible that these patients might have had previous H. pylori infection which had been eradicated.

This was a retrospective study and is limited by the lack of systemic and consistent patient evaluations. As a result, we may have underestimated the true prevalence of gastric adenomas; however, it should be noted that the prevalence of gastric adenomas in our study is similar to the previous Western experience [4]. Additionally, the number of patients with gastric adenomas in our study is relatively small; and therefore, the study may be underpowered to reveal true differences in clinical risk factors and outcomes.

## Conclusion

Gastric adenomas are common in patients with FAP and can be difficult to identify endoscopically. Endoscopists should have a high degree of suspicion for gastric adenomas in these patients and a low threshold for biopsy. Given the benign course described and that malignant transformation is rare in Western FAP patients, conservative management with endoscopic therapy and surveillance is recommended.

## Abbreviations

APC: Adenomatous polyposis coli; EGD: Esophagogastroduodenoscopy; FAP: Familial adenomatous polyposis; FGP: Fundic gland polyps; HGD: High-grade dysplasia; NSAID: Nonsteroidal anti-inflammatory drugs; PPI: Proton pump inhibitors.

## Competing interests

The authors have no competing interests to disclose.

## Authors’ contributions

SN: conception and design, collection of data, analysis and interpretation of data, drafting of the article. LAB: conception and design, critical revision of the article for important intellectual content. RIH: conception and design, critical revision of the article for important intellectual content. MK: analysis and interpretation of data, collection of images, critical revision of the article for important intellectual content. MER: collection of data, critical revision of the article for important intellectual content. DLR-J: conception and design, collection of data, collection of images, critical revision of the article for important intellectual content. All authors approved the final version of this paper.
